# Essential Role of NMDA Receptor Channel ε4 Subunit (GluN2D) in the Effects of Phencyclidine, but Not Methamphetamine

**DOI:** 10.1371/journal.pone.0013722

**Published:** 2010-10-28

**Authors:** Yoko Hagino, Shinya Kasai, Wenhua Han, Hideko Yamamoto, Toshitaka Nabeshima, Masayoshi Mishina, Kazutaka Ikeda

**Affiliations:** 1 Division of Psychobiology, Tokyo Institute of Psychiatry, Tokyo, Japan; 2 Comparative Cognitive Science Institutes and Department of Chemical Pharmacology, Graduate School of Pharmaceutical Sciences, Meijo University, Nagoya, Japan; 3 Department of Molecular Neurobiology and Pharmacology, Graduate School of Medicine, The University of Tokyo, Tokyo, Japan; Chiba University Center for Forensic Mental Health, Japan

## Abstract

Phencyclidine (PCP), a noncompetitive *N*-methyl-D-aspartate (NMDA) receptor antagonist, increases locomotor activity in rodents and causes schizophrenia-like symptoms in humans. Although activation of the dopamine (DA) pathway is hypothesized to mediate these effects of PCP, the precise mechanisms by which PCP induces its effects remain to be elucidated. The present study investigated the effect of PCP on extracellular levels of DA (DA_ex_) in the striatum and prefrontal cortex (PFC) using *in vivo* microdialysis in mice lacking the NMDA receptor channel ε1 or ε4 subunit (GluRε1 [GluN2A] or GluRε4 [GluN2D]) and locomotor activity. PCP significantly increased DA_ex_ in wildtype and GluRε1 knockout mice, but not in GluRε4 knockout mice, in the striatum and PFC. Acute and repeated administration of PCP did not increase locomotor activity in GluRε4 knockout mice. The present results suggest that PCP enhances dopaminergic transmission and increases locomotor activity by acting at GluRε4.

## Introduction

Phencyclidine (PCP) is a drug of abuse that causes psychosis resembling both the positive (e.g., hallucinations, paranoia) and negative (e.g., emotional withdrawal, motor retardation) signs of schizophrenia in humans [1]. Acute administration of PCP to rodents produces increases in locomotor activity, stereotypy, and ataxia [2], [3]. Repeated PCP administration produces sensitization of locomotor activity, rearing, and stereotypy but tolerance to ataxia [3]–[5]. PCP acts as a noncompetitive antagonist of the *N*-methyl-D-aspartate (NMDA) excitatory amino acid receptor [6]–[8]. Additionally, high doses of PCP block dopamine (DA) reuptake [1], [9]–[11]. Similar to PCP, amphetamine (AMPH) and its derivative methamphetamine (METH) produce behavioral sensitization to locomotor activity, rearing, and stereotypy when they are repeatedly administered [12], [13]. Amphetamine and METH facilitate dopaminergic neurotransmission via a number of mechanisms [14], including DA efflux by reverse transport through the dopamine transporter (DAT) [15]–[18], inhibition of DA uptake [19]–[21], and inhibition of monoamine oxidase (MAO) activity [22]–[24].

The NMDA receptor channel subunit family is composed of seven subunits—GluRζ (GluN1), GluRε1-4 (GluN2A-D), and GluRχ1, 2 (GluN3A, B)—which are all products of separate genes [25]. In the rodent and human brains, GluRε1 and GluRε2 are predominant subunits expressed in the forebrain. GluRε3 is expressed largely in cerebellar granule cells and selectively in several other brain regions. GluRε4 is expressed in the diencephalon and midbrain and is more prominent during early development [26]. Highly active NMDA receptor channels are produced when the GluRζ subunit is expressed together with one of the four GluRε subunits in *Xenopus* oocytes and mammalian cells [27]–[30]. Four GluRε subunits are major determinants of the functional properties of NMDA receptor channels [31]. Noncompetitive NMDA receptor antagonists (i.e., PCP, ketamine, and SKF-10,047) block the four GluRε/GluRζ channels to similar extents in *Xenopus* oocytes [32]. Gene-targeting techniques provide an efficient method for clarifying the distinct functions of these NMDA receptor channel subunits. GluRε1 knockout mice display increased locomotor activity, whereas GluRε4 knockout mice exhibit reduced locomotor activity in a novel environment [33]–[36]. GluRε3 knockout mice show few apparent deficits [37]–[39]. Investigating the physiological functions of GluRζ or GluRε2 knockout mice, in contrast, is nearly impossible because these two mutants die shortly after birth [40]–[42].

To clarify the contributions of NMDA receptor channel subunits in the PCP-induced increases in extracellular levels of dopamine (DA_ex_) and locomotor responses, we investigated the effects of METH and PCP on DA_ex_ in the striatum and prefrontal cortex (PFC) using *in vivo* microdialysis and measuring locomotor activity in GluRε1 knockout (GluRε1^−/−^) and GluRε4 knockout (GluRε4^−/−^) mice.

## Results

### Baseline DA_ex_ in the striatum and PFC in GluRε1^−/−^ and GluRε4^−/−^ mice

Baseline DA_ex_ was not different between wildtype, GluRε1^−/−^, and GluRε4^−/−^ mice in the striatum (one-way analysis of variance [ANOVA]: *F*_2,67_ = 0.412, *p* = 0.664) and PFC (one-way ANOVA: *F*_2,59_ = 1.025, *p* = 0.365). Mean baseline DA_ex_ in the striatum was 51.89±3.57 fmol/10 μl (*n* = 27) for wildtype, 49.35±5.35 fmol/10 μl (*n* = 19) for GluRε1^−/−^, and 46.75±3.93 fmol/10 μl (*n* = 24) for GluRε4^−/−^ mice. Mean baseline DA**_ex_** in the PFC was 1.29±0.20 fmol/10 μl (*n* = 23) for wildtype, 1.59±0.30 fmol/10 μl (*n* = 20) for GluRε1^−/−^, and 1.10±0.21 fmol/10 μl (*n* = 19) for GluRε4^−/−^ mice.

### Effects of acute METH administration on DA_ex_ in the striatum and PFC in GluRε1^−/−^ and GluRε4^−/−^ mice

**Figure 1:**
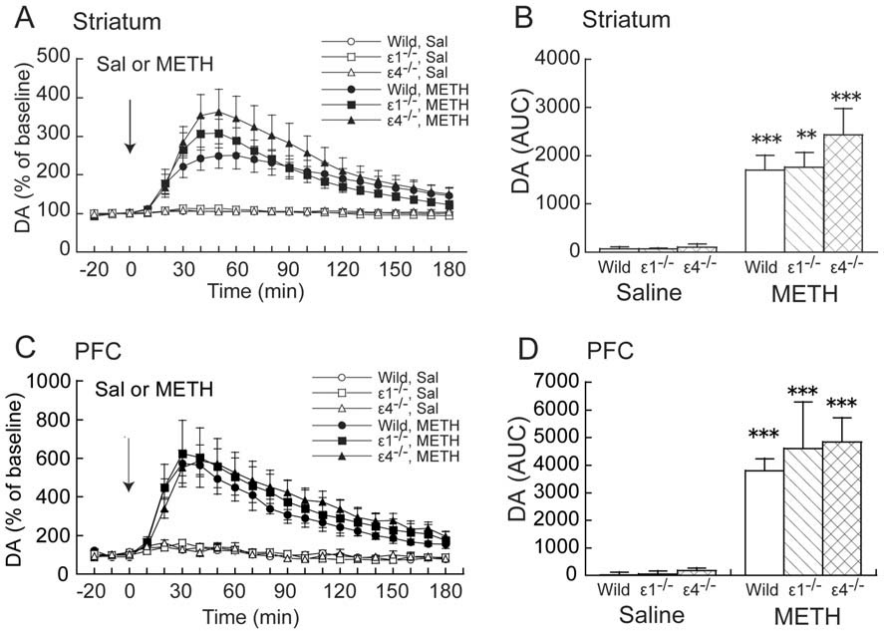
Effects of acute METH on DA_ex_ in the striatum and PFC in wildtype, GluRε1^−/−^, and GluRε4^−/−^ mice. (**A**, **C**) Temporal pattern of DA_ex_ before and after s.c. saline (Sal) or METH (1 mg/kg) injection. The arrows indicate the drug injection time. Each point represents the mean ± SEM of the percentage of DA_ex_ baseline. (**B**, **D**) Histogram representing the mean AUC ± SEM of DA_ex_ during the 180 min period after saline or METH injection (*n* = 5–9). **p<0.01, ***p<0.001, compared with saline group of the same genotype (two-way ANOVA followed by Fisher's PLSD *post hoc* test).

Methamphetamine (1 mg/kg) markedly increased DA_ex_ in the striatum and PFC in wildtype, GluRε1^−/−^, and GluRε4^−/−^ mice (Fig. 1A, C). Two-way ANOVA (drug × genotype) of DA_ex_, measured as the area-under-the-curve (AUC) calculated during a 180 min posttreatment period, revealed a significant effect of drug (*F*_1,39_ = 47.418, *p*<0.001) but not genotype (*F*_2,39_ =  0.889, *p* = 0.419) and no significant drug × genotype interaction (*F*_2,39_ = 0.739, *p* = 0.484) in the striatum (Fig. 1B). Similarly, in the PFC, two-way ANOVA (drug × genotype) of AUC values revealed a significant effect of drug (*F*_1,31_ = 48.784, *p*<0.001) but not genotype (*F*_2,31_ = 0.320, *p* = 0.728) and no significant drug × genotype interaction (*F*_2,31_ = 0.201, *p* = 0.819) (Fig. 1B).

### Effects of acute PCP administration on DA_ex_ in the striatum and PFC in GluRε1^−/−^ and GluRε4^−/−^ mice

**Figure 2:**
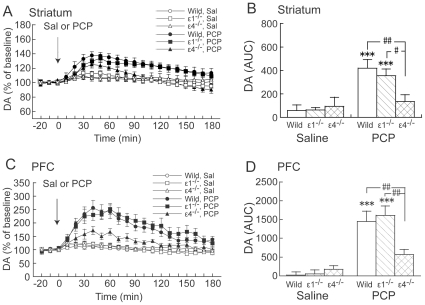
Effects of acute PCP on DA_ex_ in the striatum and PFC in wildtype, GluRε1^−/−^, and GluRε4^−/−^ mice. (**A**, **C**) Temporal pattern of DA_ex_ before and after s.c. saline (Sal) or PCP (3 mg/kg) injection. The arrows indicate the drug injection time. Each point represents the mean ± SEM of the percentage of DA_ex_ baseline. (**B**, **D**) Histogram representing the mean AUC ± SEM of DA_ex_ during the 180 min period after saline or PCP injection (*n* = 5–11). ***p<0.001, compared with saline group of the same genotype; ^#^ *p*<0.05, ^##^ *p*<0.01, comparisons between genotypes in the same drug treatment (two-way ANOVA followed by Fisher's PLSD *post hoc* test).

Phencyclidine (3 mg/kg) markedly increased DA_ex_ in wildtype and GluRε1^−/−^ mice, but not in GluRε4^−/−^ mice, in the striatum and PFC (Fig. 2A, C). Two-way ANOVA (drug × genotype) of AUC values revealed a significant effect of drug (*F*_1,39_ = 17.201, *p*<0.001) but not genotype (*F*_2,39_ = 2.012, *p* = 0.147) in the striatum and a significant drug × genotype interaction (*F*_2,39_ = 3.314, *p* = 0.047) (Fig. 2B). *Post hoc* comparisons revealed that the effect of PCP on DA_ex_ in GluRε4^−/−^ mice was significantly less compared with wildtype and GluRε1^−/−^ mice (*p* = 0.002 and 0.03, respectively; Fisher's Protected Least Significant Difference [PLSD] *post hoc* test) in the striatum (Fig. 2B). In the PFC, two-way ANOVA (drug × genotype) of AUC values revealed a significant effect of drug (*F*_1,37_ = 35.215, *p*<0.001) but not genotype (*F*_2,37_ = 1.969, *p* = 0.154) and a significant drug × genotype interaction (*F*_2,37_ = 3.326, *p* = 0.047) (Fig. 2D). *Post hoc* comparisons revealed that the effect of PCP on DA_ex_ in GluRε4^−/−^ mice was significantly less compared with wildtype and GluRε1^−/−^ mice (*p* = 0.007 and 0.003, respectively; Fisher's PLSD *post hoc* test) in the PFC (Fig. 2D).

### Locomotor activity in GluRε1^−/−^ and GluRε4^−/−^ mice in a novel environment

**Figure 3:**
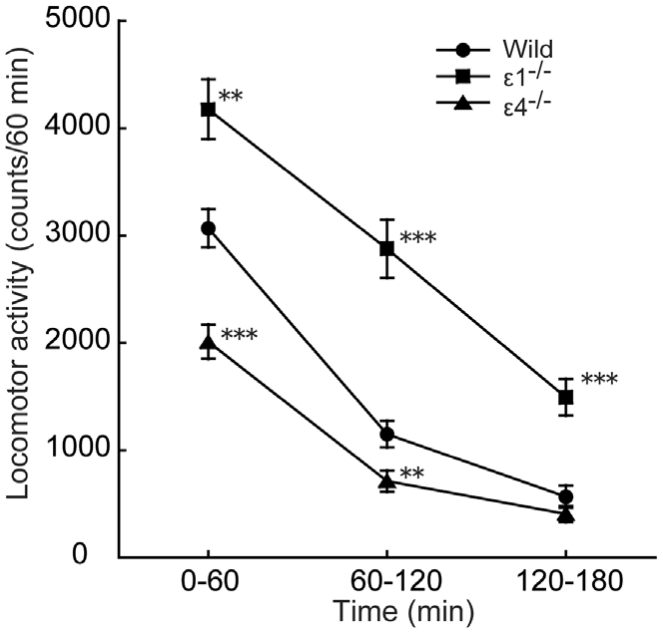
Locomotor activity in wildtype, GluRε1^−/−^, and GluRε4^−/−^ mice in a novel environment. Locomotor activity was measured for 180 min. Each point represents the mean ± SEM (*n* = 34–50). *p<0.05, **p<0.01, ***p<0.001, compared with wildtype mice (one-way ANOVA followed by Fisher's PLSD *post hoc* test).

Locomotor activity in a novel environment was different between wildtype, GluRε1^−/−^, and GluRε4^−/−^ mice during the habituation period (mixed-design ANOVA: genotype, *F*_2,123_ = 35.423, *p*<0.0001; time, *F*_2,123_ = 486.554, *p*<0.0001; genotype × time, *F*_4,123_ = 15.337, *p*<0.0001) (Fig. 3). Locomotor activity in a novel environment during the 60 min period increased in GluRε1^−/−^ mice (*p* = 0.0002, unpaired *t*-test) but decreased in GluRε4^−/−^ mice (*p*<0.0001, Student's *t*-test) compared with wildtype mice. GluRε1^−/−^ mice did not habituate during the 180 min period compared with wildtype mice (*p*<0.0001, Student's *t*-test).

### Effects of acute administration of METH and PCP on locomotor activity in GluRε1^−/−^ and GluRε4^−/−^ mice

**Figure 4:**
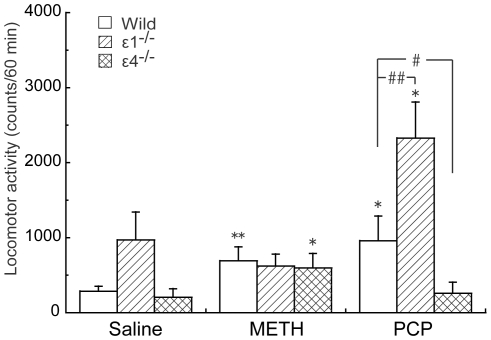
Effects of acute METH and PCP on the locomotor activity in GluRε1^−/−^ and GluRε4^−/−^ mice. Locomotor activity after acute saline, METH (1 mg/kg), or PCP (3 mg/kg) administration (*n* = 10–25). *p<0.05, **p<0.01, compared with saline (Student's *t*-test); ^#^ *p*<0.05, ^##^ *p*<0.01, compared with wildtype (Student's *t*-test).

Two-way ANOVA (drug × genotype) of locomotor activity data during the 60 min period revealed significant effects of drug (*F*_2,155_ = 8.646, *p* = 0.0002) and genotype (*F*_2,155_ = 11.769, *p*<0.0001) and a significant drug × genotype interaction (*F*_4,155_ = 5.734, *p* = 0.0002) (Fig. 4). Methamphetamine (1 mg/kg) significantly increased locomotor activity during the 60 min period after the METH injection in wildtype mice (*p* = 0.002, Student's *t*-test) and GluRε4^−/−^ mice (*p* = 0.0004, Student's *t*-test) compared with saline. However, METH (1 mg/kg) did not increase locomotor activity during the 60 min period after the METH injection in GluRε1^−/−^ mice (*p* = 0.411, Student's *t*-test) compared with saline.

Phencyclidine (3 mg/kg) significantly increased locomotor activity during the 60 min period after the PCP injection in wildtype mice (*p* = 0.008, Student's *t*-test) and GluRε1^−/−^ mice (*p* = 0.045, Student's *t*-test) compared with saline treatment. However, PCP (3 mg/kg) did not increase locomotor activity in GluRε4^−/−^ mice (*p* = 0.142, unpaired *t*-test) compared with saline treatment.

### Effects of repeated administration of METH and PCP on locomotor activity in GluRε1^−/−^ and GluRε4^−/−^ mice

**Figure 5:**
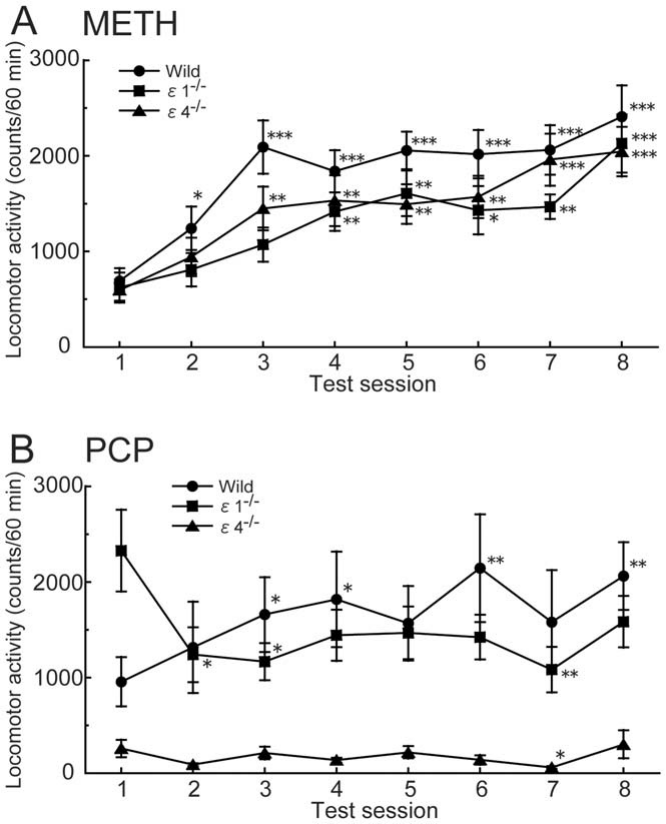
Effects of repeated METH and PCP on the locomotor activity in GluRε1^−/−^ and GluRε4^−/−^ mice. Changes in response to repeated administration of (**A**) METH (1 mg/kg) or (**B**) PCP (3 mg/kg) (*n* = 15–25). *p<0.05, **p<0.01, ***p<0.001, compared with Session 1 of the same genotype (paired *t*-test). Each point represents total locomotor activity (mean ± SEM) during the 60 min period after METH or PCP injection.

Mixed-design ANOVA of locomotor activity data during the 60 min period after the METH injection from Session 1 to 8 revealed significant effects of genotype (*F*_2,385_ = 3.350, *p* = 0.042) and session (*F*_7,385_ = 16.091, *p*<0.0001) but no significant genotype × session interaction (*F*_14,385_ = 0.611, *p* = 0.857) (Fig. 5A). Chronic METH (1 mg/kg) injections increased locomotor activity in wildtype (*p*<0.0001, paired *t*-test), GluRε1^−/−^ (*p* = 0.0007, paired *t*-test), and GluRε4^−/−^ mice (*p* = 0.0001, paired *t*-test) in Session 1 compared with Session 8.

Mixed-design ANOVA of locomotor activity data during the 60 min period after the PCP injection revealed a significant effect of genotype (*F*_2,455_ = 11.318, *p*<0.0001) but not session (*F*_7,455_ = 1.443, *p* = 0.186) and a significant genotype × session interaction (*F*_14,455_ = 2.368, *p* = 0.0035) (Fig. 5B). Phencyclidine-induced hyperactivity was significantly greater in Session 8 than Session 1 in wildtype mice (*p* = 0.006, paired *t*-test). Repeated PCP (3 mg/kg) administration did not increase locomotor activity in GluRε1^−/−^ mice (*p* = 0.121, paired *t*-test) and GluRε4^−/−^ mice (*p* = 0.605, paired *t*-test) in Session 1 compared with Session 8.

## Discussion

The present study showed that PCP-induced increases in DA_ex_ in the striatum and PFC and locomotor activity were absent in GluRε4^−/−^, but present in GluRε1^−/−^, mice, indicating that GluRε4 plays an important role in PCP-increased DA_ex_ and locomotor activity. Phencyclidine exerts psychotomimetic effects, whereas another NMDA receptor antagonist, MK-801, exerts no clear psychotomimetic effects in humans [43]. Interestingly, whereas MK-801 suppresses GluRε3/GluRζ1 and GluRε4/GluRζ1 channels more weakly than GluRε1/GluRζ1 and GluRε2/GluRζ1 channels, PCP blocks the four GluRε/GluRζ channels to similar extents in *Xenopus* oocytes [32]. The absence of psychotomimetic effects of MK-801 may be attributable to its weak ability of blocking the GluRε4/GluRζ1 channel.

Systemic administration of PCP reportedly increases DA_ex_ in the striatum and PFC [44]–[49]. Similarly, PCP (3 mg/kg) increased DA_ex_ in wildtype and GluRε1^−/−^ mice in the present study. However, PCP failed to increase DA_ex_ in the striatum and PFC in GluRε4^−/−^ mice. Phencyclidine is known to be a DA reuptake blocker and a noncompetitive NMDA antagonist [9]–[11]. It inhibits DA uptake by binding to the DAT at doses approximately 10-fold greater than those at which it binds to NMDA receptor channels [1]. Phencyclidine at the low dose used in the present study appears to have few effects on the DAT. Furthermore, no PCP-induced increases in DA_ex_ in GluRε4^−/−^ mice that possess an intact DAT gene indicates that PCP increases DA_ex_ not via DAT inhibition but via blockade of NMDA receptor channels. The present results support the hypothesis that GluRε4 is an important determinant of increased DA_ex_ induced by PCP. Acute administration of METH increased DA_ex_ in the striatum and PFC in wildtype, GluRε1^−/−^, and GluRε4^−/−^ mice. No differences in DA_ex_ increases were found between genotypes. The similar DA_ex_ increases among these mice in response to acute METH challenge suggest that increased DA_ex_ occurs independently of GluRε1^−/−^ and GluRε4^−/−^.

Locomotor activity in a novel environment is reportedly high in GluRε1^−/−^ mice [34], [36] and low in GluRε4^−/−^ mice [33], [35]. Consistent with these findings, increased locomotor activity in GluRε1^−/−^ mice and reduced locomotor activity in GluRε4^−/−^ mice were observed in the present study. GluRε1^−/−^ mice did not habituate during the 180 min period compared with wildtype mice. Interestingly, acute METH administration decreased locomotor activity in GluRε1^−/−^ mice. Hyperactivity and a paradoxical response to METH suggest that GluRε1^−/−^ mice may be an animal model of attention-deficit/hyperactivity disorder.

Psychostimulants, such as METH and PCP, increase locomotor activity [2], [3], [12], [13]. In GluRε4^−/−^ mice, acute METH administration increased locomotor activity, but PCP did not. Acute PCP administration increased locomotor activity in wildtype and GluRε1^−/−^ mice, but not in GluRε4^−/−^ mice. The absence of locomotor-stimulating effects of PCP in GluRε4^−/−^ mice indicates that locomotor responses to PCP require the GluRε4 subunit.

Repeated administration of PCP produces sensitization to its locomotor-stimulating effects in wildtype mice. In GluRε4^−/−^ mice, locomotor activity did not increase after repeated PCP treatment. Acute PCP did not increase locomotor activity, and repeated PCP did not produce sensitization to the locomotor-stimulating effects of PCP in GluRε4^−/−^ mice. The GluRε4 subunit appears to be necessary for behavioral sensitization to occur in response to repeated PCP administration. A previous study demonstrated that acute PCP treatment increased locomotor activity in wildtype and GluRε1^−/−^ mice. Chronic PCP treatment at a low dose (3 mg/kg/day) for 7 days produced sensitization to the locomotor-stimulating effects of PCP in wildtype mice, but not in GluRε1^−/−^ mice [50]. The present study confirmed that repeated PCP administration (3 mg/kg/day) did not produce sensitization during Session 8 in GluRε1^−/−^ mice. Repeated METH administration produced behavioral sensitization in wildtype, GluRε1^−/−^, and GluRε4^−/−^ mice. The development of sensitization in GluRε1^−/−^ and GluRε4^−/−^ mice was delayed compared with wildtype mice. The noncompetitive NMDA receptor antagonist MK-801 has been shown to block the development of behavioral sensitization to AMPH and METH [51]–[54]. Molecular and cellular adaptive changes during chronic drug exposure are hypothesized to lead to the development of sensitization. Our findings support the hypothesis that adaptive changes through NMDA receptor channels play a role in the development of locomotor sensitization to METH.

Schizophrenia is a disease that has been hypothesized to be associated with hyperfunction of the dopaminergic neuronal system and dysfunction of glutamatergic transmission [55], [56]. Administration of PCP to normal humans induces symptoms similar to those of schizophrenia [57]. This finding has been replicated over the years, and PCP has been shown to exacerbate the primary symptoms of schizophrenic patients [56]. Phencyclidine-treated animals have been used as an animal model of schizophrenia, and the amelioration of hyperlocomotion in these animals has been used as a screening test to assess the efficacy of antipsychotic drugs [58], [59]. GluRε4 immunoreactivity and protein expression increase in the frontal cortex following repeated PCP treatment, whereas GluRε1 immunoreactivity and protein expression are not altered in rats [60]. Furthermore, polymorphisms of several genes known to interact with NMDA receptor channels are related to altered risk for schizophrenia, and psychotic patients display changes in the levels of mRNA encoding NMDA receptors [61]. Interestingly, Makino *et al.* reported that the GluRε4 gene locus is a possible genomic region that contributes to schizophrenia susceptibility in a Japanese population [62]. In the present study, we first demonstrated that deletion of GluRε4 abolished PCP-induced hyperlocomotion and potentiated the increases in DA_ex_ in mice. Our data and previous findings suggest that GluRε4 might be a potential target for antipsychotic drug development.

Although NMDA receptor channels are highly expressed in adult brains, adult GluRε4 expression is very limited [26]. GluRε4 is expressed in the substantia nigra compacta (SNc), subthalamic nucleus, globus pallidus, and ventral pallidum in adult rats [63]. Jones and Gibb reported that functional GluRε2 and GluRε4 subunits form somatic NMDA receptors, possibly as triheteromeric receptors, whereas no somatic GluRε1 subunits are present in SNc dopaminergic neurons in rats aged postnatal day 14 [64]. A small subset of NMDA receptor channels (i.e., channels containing GluRε4) may be implicated in the effects of PCP on DA_ex_ and locomotor activity. This possibility is consistent with the lack of psychotic effects of ifenprodil, a selective blocker of NMDA receptor channels containing GluRε2, which is highly expressed in adult brains. Additionally, GluRε4 is highly expressed in the brain during development [26], suggesting that GluRε4 knockout during the developmental stage may alter neuronal function in the adult brain. Although the expression of the genes related to dopaminergic signaling pathways are not altered in GluRε4^−/−^ mice during adulthood (see Table S1), other developmental changes may alter the effects of PCP in GluRε4^−/−^ mice. Further studies of synapses, neurons, and neuronal networks regulated by GluRε4 and developmental changes in neuronal function in GluRε4^−/−^ mice may lead to a better understanding of the mechanisms underlying PCP-induced psychosis and schizophrenia.

## Materials and Methods

### Ethics statement

The experimental procedures and housing conditions were approved by the Institutional Animal Care and Use Committee (Animal Experimentation Ethics Committee of Tokyo Institute of Psychiatry, Approval ID: 22-2), and all animal were cared for and treated humanely in accordance with our institutional animal experimentation guidelines.

### Animals

Wildtype and GluRε1^−/−^ or GluRε4^−/−^ mouse littermates from crosses of heterozygous/heterozygous GluRε1 or GluRε4 knockout mice, respectively, on a C57BL/6 genetic background [33], [65] served as subjects. Naive adult mice were housed in an animal facility maintained at 22±2°C and 55±5% relative humidity under a 12 h/12 h light/dark cycle with lights on at 8:00 am and off at 8:00 pm. Food and water were available *ad libitum*. In the behavioral experiments, 13- to 23-week-old male mice were used. In the microdialysis experiments, 10- to 24-week-old male and female mice were used.

### Surgery

Microdialysis probes were stereotaxically implanted in mice under sodium pentobarbital anesthesia (50 mg/kg, intraperitoneally) in the striatum (anterior, +0.6 mm; lateral, +1.8 mm; ventral, −4.0 mm from bregma) or PFC (anterior, +2.0 mm; lateral, +0.5 mm; ventral −3.0 mm from bregma), according to the atlas of Franklin and Paxinos [66]. The probe tip was constructed with a regenerated cellulose membrane (outer diameter, 0.22 mm; membrane length, 2 mm; Eicom, Kyoto, Japan). All dialysis probe placements were verified histologically at the completion of the experiment.

### Microdialysis and analytical procedures

Twenty-four hours after implantation, the dialysis experiments were performed in freely moving animals. Ringer's solution (145 mM NaCl, 3 mM KCl, 1.26 mM CaCl_2_, and 1 mM MgCl_2_, pH 6.5) was perfused at a constant flow rate of 1 µl/min. Perfusates were directly injected into the high-performance liquid chromatography system every 10 min using an autoinjector (EAS-20; Eicom). Dialysate DA was separated using a reverse-phase ODS column (PP-ODS; Eicom) and detected with a graphite electrode (HTEC-500; Eicom). The mobile phase consisted of 0.1 M phosphate buffer (pH 5.5) containing 500 mg/l sodium decanesulfonate, 50 mg/l EDTA, and 1% methanol. Perfusion was initiated 180 min prior to the collection of baseline samples. Baseline levels of DA_ex_ were obtained from the average concentrations of three consecutive samples when they were stable. The DA detection limit of the assay was 0.3 fmol/sample with a signal-to-noise ratio of 2.

### Locomotor activity measurements

Each mouse were exposed to an illuminated chamber (30×40×25 cm) at an ambient temperature of 22±2°C, and locomotor activity was measured with Supermex (Muromachi Kikai, Tokyo, Japan), a sensor monitor mounted above the chamber. In this system, a sensor detects the radiated body heat of an animal [67]. This measurement system can detect changes in heat across multiple zones of the chamber and count all horizontal movements. All counts were automatically summed and recorded every 5 min. After a 180 min habituation period, METH or PCP was administered subcutaneously (s.c.), and locomotor activity was monitored continuously for 180 min.

### Drugs

Drugs were dissolved in saline and administered s.c. in a volume of 10 ml/kg. In the microdialysis experiment, saline, METH (1 mg/kg), or PCP (3 mg/kg) was administered after establishing a stable baseline, and the dialysate was continuously collected for 180 min. In the acute behavioral experiments, saline, freshly prepared METH (1 mg/kg; Dainippon Sumitomo Pharma, Osaka, Japan), or PCP (3 mg/kg; Shionogi Pharmaceutical Co. Ltd., Osaka, Japan) was administered. In the repeated behavioral experiments, METH (1 mg/kg) or PCP (3 mg/kg) was administered repeatedly at 2 or 3 day intervals for a total of seven injections. One week after withdrawal, METH or PCP challenge injections were administered as described above.

### Statistical analysis

DA_ex_ responses to drugs are expressed as a percentage of baseline. The AUC of DA_ex_ during the 180 min period after drug administration was calculated as the effects of the drugs. Area-under-the-curve values of all groups were analyzed using two-way ANOVA. Individual *post hoc* comparisons were performed with Fisher's PLSD test. The responses to acute administration were analyzed using Student's *t*-test, one-way ANOVA, or two-way ANOVA. To evaluate behavioral sensitization, the response to drugs in Session 8 was compared with the response to the first drug injection (Session 1) in the same animal using a paired *t*-test or mixed-design ANOVA. Values of *p*<0.05 were considered statistically significant. Data were analyzed using Statview J5.0 software (SAS Institute, Cary, NC, USA).

## Supporting Information

Table S1 Striatal gene expression in wildtype and GluRε4-/- mice.
(0.03 MB DOC)
