# Supplementary material for: Essential Role of NMDA Receptor Channel ε4 Subunit (GluN2D) in the Effects of Phencyclidine, but Not Methamphetamine
Source: PLoS One. 2010 Oct 28;5(10):e13722. doi: 10.1371/journal.pone.0013722 (PMC2965660; doi:10.1371/journal.pone.0013722)
Supplement: Table S1 — Striatal gene expression in wildtype and GluRε4-/- mice. (0.03 MB DOC) [file pone.0013722.s001.doc]

Table S1. Striatal gene expression in wildtype and GluRε4^-/-^ mice.

| Gene | Wildtype | GluRε4^-/-^ |
| --- | --- | --- |
| dopamine receptor D1A (*Drd1a*)  dopamine receptor 2 (*Drd2*)  dopamine receptor 3 (*Drd3*)  dopamine receptor 4 (*Drd4*)  dopamine receptor 5 (*Drd5*)  monoamine oxidase A (*Maoa*), nuclear gene encoding mitochondrial protein  monoamine oxidase B (*Maob*), nuclear gene encoding mitochondrial protein  catechol-*O*-methyltransferase domain containing 1 (*Comtd1*)  dopamine β-hydroxylase (*Dbh*)  tyrosine hydroxylase (*Th*)  solute carrier family 6 (neurotransmitter transporter, noradrenalin), member 2 (*Slc6a2*)  solute carrier family 6 (neurotransmitter transporter, dopamine), member 3 (*Slc6a3*)  solute carrier family 6 (neurotransmitter transporter, serotonin), member 4 (*Slc6a4*)  solute carrier family 18 (vesicular monoamine), member 2 (*Slc18a2*) | 5155.4 ± 156.0  2030.2 ± 158.2  153.9 ± 14.5  143.3 ± 5.4  169.3 ± 8.0  162.3 ± 12.9  352.1 ± 8.9  167.2 ± 12.2  144.1 ± 14.1  195.0 ± 13.0  149.0 ± 9.4  163.2 ± 11.4  179.8 ± 10.4  167.4 ± 9.1 | 5072.8 ± 404.5  2091.4 ± 93.8  161.6 ± 7.3  149.5 ± 10.0  172.8 ± 11.2  180.1 ± 10.4  369.6 ± 20.8  175.5 ± 17.2  142.7 ± 12.5  196.5 ± 7.0  152.8 ± 8.7  175.0 ± 14.6  189.8 ± 18.5  171.8 ± 10.5 |

Background: wildtype mice, 148.6 ± 14.4; GluRε4^-/-^ mice, 150.2 ± 8.1.

**Results**

Gene expression related to dopaminergic signaling pathways was not significantly altered by GluRε4 knockout in the striatum.

**Methods**

*Tissue preparation, RNA isolation, probe labeling, and microarray hybridization*

The striata from 10 mice per genotype group were quickly dissected on ice and immediately frozen at -80°C. The striata from 10 mice per treatment group were pooled and used for the DNA array experiment.

*RNA isolation*

Total RNA was isolated with TRIzol reagent (Invitrogen, Carlsbad, CA) and purified with the RNeasy Mini Kit according to the manufacturer’s instructions (Qiagen, Valencia, CA). RNA was quantified by measuring optical density at 260 nm using a spectrophotometer (Beckman, Fullerton, CA).

*Microarray gene expression analysis*

Gene expression profiling for four sets of two RNA samples was performed using microarray platforms from Illumina (San Diego, CA). For the Illumina platform, we used the multi-sample format MouseRef-8 BeadChip with over 24,000 probes simultaneously profiling eight samples on a single chip. Briefly, 500 ng of total RNA was labeled using an Illumina TotalPrep RNA Amplification Kit (Applied Biosystems, Foster City, CA). Double-stranded cDNA was synthesized using T7-oligo (dT) primers followed by an *in vitro* transcription (IVT) reaction to amplify antisense-RNA (aRNA), whereas biotin was incorporated into the synthesized aRNA target. The biotinylated cRNA target was hybridized to the MouseRef-8 BeadChip. Hybridization, washing, and scanning were performed according to the manufacturer’s instructions following overnight hybridization. The chips were scanned using a BeadScan (Illumina) at a multiplier setting of 2. The microarray images were recorded, and gene expression data were extracted automatically according to the manufacturer’s default settings. Raw microarray intensity data were provided using differential expression algorithms (Illumina).
